# *QuickStats:* Age-Adjusted Percentage* of Adults Aged ≥18 Years Who Currently Have Asthma,^†^ by Sex and Race/Ethnicity^§^ ─ National Health Interview Survey, United States, 2017–2018^¶^

**DOI:** 10.15585/mmwr.mm6925a7

**Published:** 2020-06-26

**Figure:**
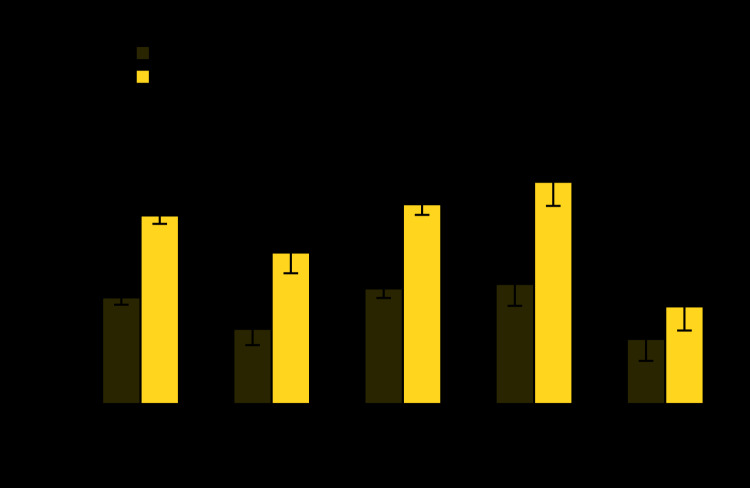
During 2017–2018, women aged ≥18 years were more likely than men (9.7% versus 5.5%) to currently have asthma. This pattern prevailed in each of the race/ethnicity groups: Hispanic adults (7.8% versus 3.9%); non-Hispanic white adults (10.3% versus 5.9%); non-Hispanic black adults (11.4% versus 6.2%); and non-Hispanic Asian adults (5.0% versus 3.3%). Non-Hispanic white and non-Hispanic black men were more likely to currently have asthma than were Hispanic and non-Hispanic Asian men. The same pattern existed among women.

**Source:** National Health Interview Survey, 2017–2018 data. https://www.cdc.gov/nchs/nhis.htm.

